# The impact of thigmotaxis deprivation on the development of the German cockroach (*Blattella germanica*)

**DOI:** 10.1016/j.isci.2022.104802

**Published:** 2022-07-20

**Authors:** Yun-Ru Chen, De-Wei Li, Hsin-Ping Wang, Shih-Shun Lin, En-Cheng Yang

**Affiliations:** 1 Department of Entomology, National Taiwan University, Taipei, Taiwan, R.O.C.; 2 Institute of Biotechnology, National Taiwan University, Taipei, Taiwan, R.O.C.

**Keywords:** Entomology, Molecular mechanism of behavior

## Abstract

Thigmotaxis is required in small animals. In this study, we examined how the shelter angle affects the development of German cockroaches, *Blattella germanica*. Groups and individual cockroaches showed a strong preference for shelters with an angle of ≤40° after 15 min or 24 h in shelter-selection trials. For cockroaches that developed in 90/180-degree shelters, survival and fecundity were low, and the nymphal stage lasted longer. Post-molting transcriptomes of second- and sixth-instar nymphs were analyzed at 12 h and 2 days post-molting. Upregulation was observed in genes related to ATP metabolism and cellular amide metabolism. Chitin-based cuticle development and postembryonic development-related genes were downregulated. The stress responses of cockroaches that developed in shelters with angles of 90° were similar to those of gregarious cockroaches experiencing social isolation. For German cockroaches, environmental tactile stimuli are crucial to development and homeostasis.

## Highlights

• German cockroaches tended to prefer shelters with an angle of ≤40
• Both fecundity and survival are low in 90°/180° developed cockroaches
• Genes for cuticle development were down-regulated in 90°/180° developed cockroaches
• German cockroaches require a shelter with an angle of ≤40° for development

## Graphical abstract

**Figure:**
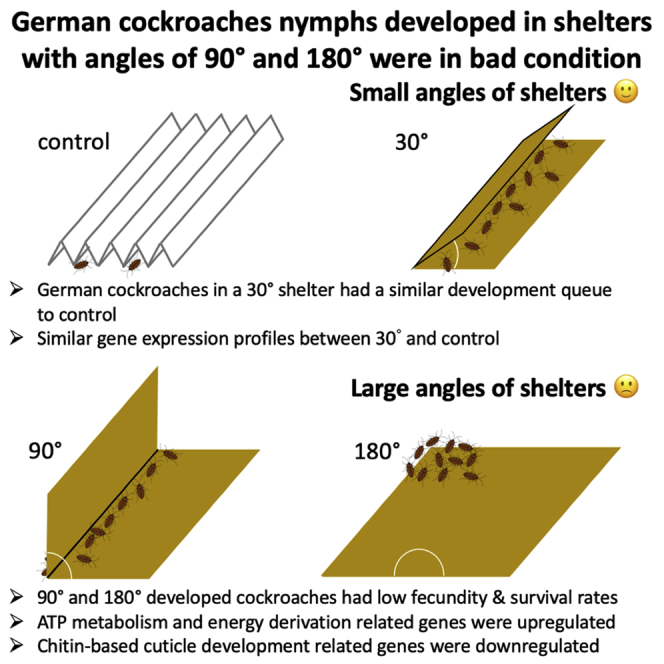

## Introduction

Thigmotaxic behavior occurs in both vertebrates and invertebrates, especially when individuals encounter a new environment, and is thought to be associated with shelter-seeking behavior to avoid danger from predators (Treit and Fundytus, 1988; Simon et al., 1994; Durier and Rivault, 2003; Jeanson et al., 2003; Besson and Martin, 2004; Sharma et al., 2009; Schnörr et al., 2012). In cockroaches and other insects, positive thigmotaxic behavior is known to be associated with foraging and hiding behavior to escape predators or other threats, and the degree of thigmotaxis also affects the fleeing behavior of cockroaches (Salazar et al., 2018).

Cockroaches prefer to hide in narrow gaps that can provide sufficient thigmotaxic feedback, and they gather environmental information using multiple sensory modalities, including olfactory, gustatory, and tactile input, to guide their movement decisions (Hebets and Papaj, 2005; Partan and Marler, 2005; Uzsák et al., 2014). The antennae are the major receptive organs used by insects to receive chemical or mechanical stimuli for social communication and thigmotaxic and environmental stimulation (Minnich, 1929; Schneider, 1964; Callahan, 1975). In cockroaches, the antennae can also be used to evaluate the shape/structure of a shelter to allow positional adjustment to ensure the best fit within the space (Jayaram and Full, 2016). A single antenna of an American cockroach is sufficient to distinguish a target object’s relative position at angles of 0°, 45°, and 90° to the horizontal plane (Okada and Toh, 2000). By identifying different geometric structures, cockroaches are able to search for appropriate shelters. Shelters not only provide protection but also potentially fulfill specific physiological requirements for cockroach development. Cockroaches that develop in areas with shelters show faster development rates, higher adult body weights, and more fertile oothecae than those that develop without shelters (Gemeno et al., 2011). Nymphs in open areas without shelters show greater aggregation behavior (Ledoux, 1945). These observations suggest that cockroaches require environmentally derived tactile stimuli, but the degree of this requirement is not yet fully understood.

With its relatively small size and short reproductive cycle, the German cockroach, *Blattella germanica*, thrives in human habitations and has become one of the most common pests worldwide (Cochran, 2009). In this study, we used German cockroaches as the experimental subjects. We hypothesized that stimuli reflecting the geometric features of the environment, especially the angles of a potential shelter, can affect their developmental homeostasis. By establishing life tables, survival rates, and gene expression profiles, we were able to understand how the angles of shelters affect the development of this species.

## Results

### Shelter selection by groups of nymphs and adult German cockroaches after 24 h

**Figure 1:**
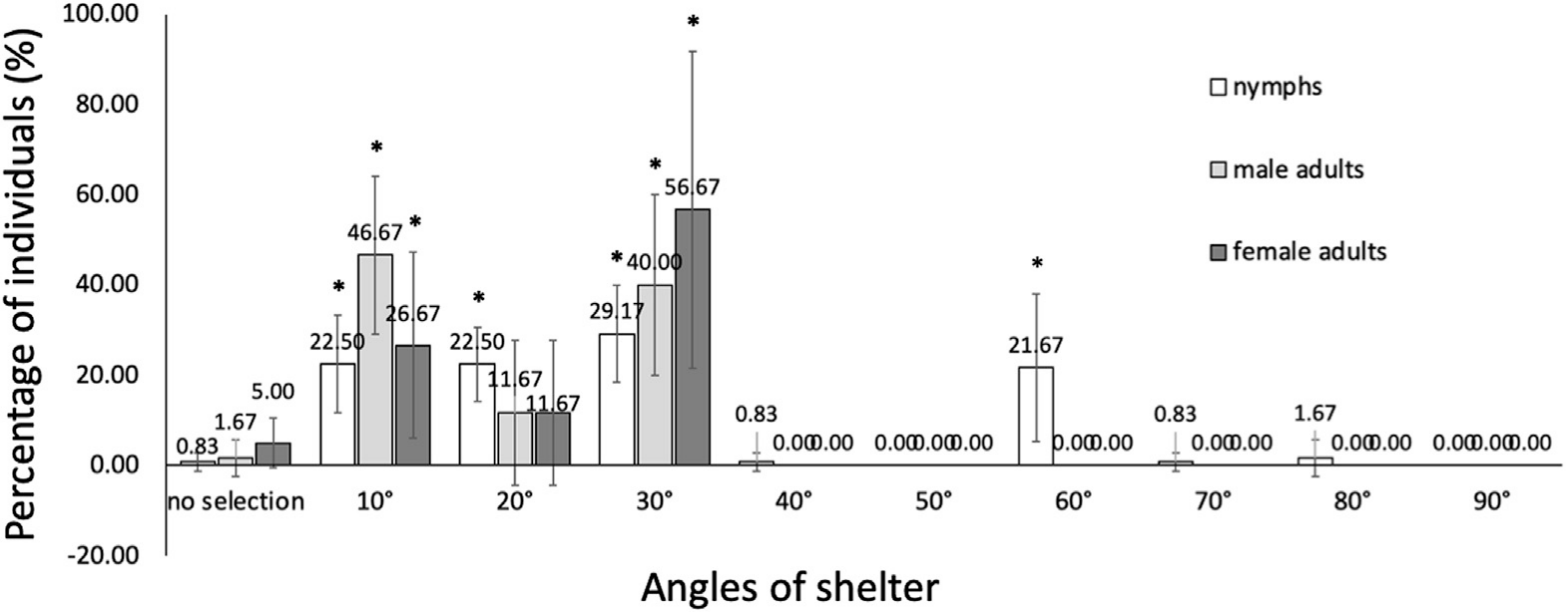
Shelter selection of groups of German cockroaches after 24 hours An acrylic cylinder with a lid (inside diameter × height = 59 × 11.5 cm; outside diameter × height = 60 × 20 cm) was designed as the test environment (the 60 cm cylinder). Copper shelters with fold angles of 10°, 20°, 30°, 40°, 50°, 60°, 70°, 80°, and 90° were evenly distributed inside the acrylic cylinder at a distance of 18 cm from the center of the cylinder. A total of 20 nymphs, 10 adult males or 10 adult females were placed at the center of the acrylic cylinder for shelter selection. The angle of the chosen copper shelter was recorded 24 h later, after which the cockroaches were removed. X axis: the angle of the copper shelter; Y axis: the percentage of cockroaches choosing a shelter. ∗p < 0.05, Student’s *t* test.

In the 24-h shelter selection assay, a long, standardized time was provided for cockroaches to explore the environment, such that their selected shelter may represent their preferences. After 24 h allotted for shelter selection, adults showed a strong preference for shelters with an angle less than or equal to 30°. A total of 56.67% of females hid in the 30° shelter, while more than 80% of males chose shelters with angles of 10 and 30° (Figure 1). In contrast to the adults, nymphs aggregated in shelters with angles of 10° (22.5%), 20° (22.5%), 30° (29.17%), and 60° (21.67%), and 3.34% of them showed no selection (stayed outside all the shelters) or chose shelters with an angle larger than 60° (Figure 1).

### Shelter selection by individual German cockroaches after 15 min

**Figure 2:**
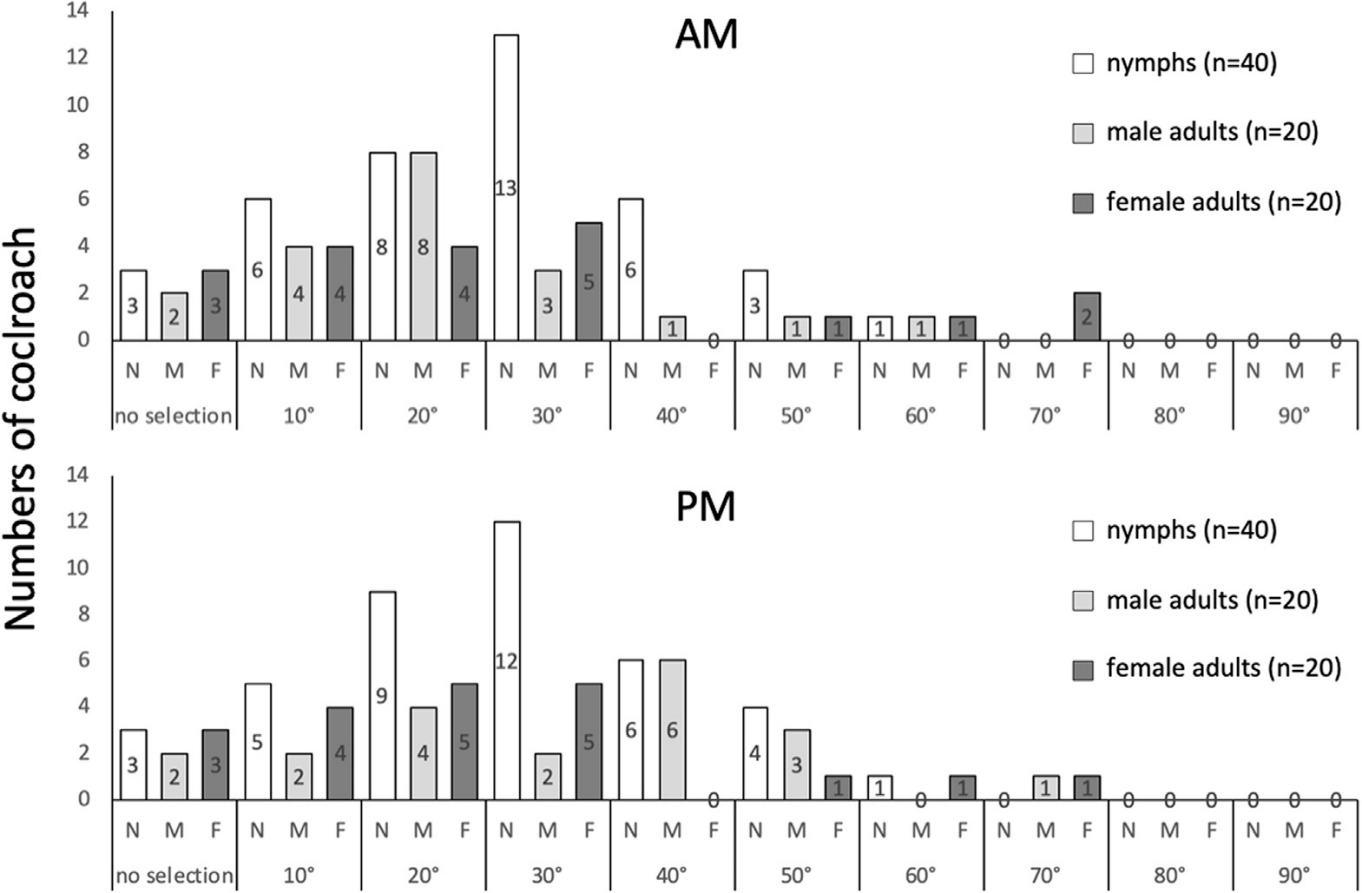
Shelter selection by individual German cockroaches after 15 minutes One nymph (N), adult male (M), or adult female (F) German cockroach at a time was placed at the center of the acrylic cylinder for shelter selection. Copper shelters with angles of 10°, 20°, 30°, 40°, 50°, 60°, 70°, 80°, and 90° were distributed evenly inside the 60 cm cylinder at a distance of 18 cm from the center of the cylinder. The angle of the chosen copper shelter was recorded 15 min later, after which the cockroach was removed. X axis: the angle of the copper shelter; Y axis: the number of cockroaches choosing a shelter.

In the 15-min shelter selection assay, the shelter preference of cockroaches may represent a quick response after entry into a new environment. After 15 min were allocated for shelter selection, many German cockroaches chose to hide in shelters with angles less than or equal to 40° during the morning and less than or equal to 50° during the afternoon (Figure 2). Males showed a strong preference for the 20° (40%) and 40° (30%) shelters during the morning and afternoon, respectively, while females showed no significant preference in the morning (p = 0.067, Kendall rank correlation coefficient, Table S2A) but showed a preference for shelter angles less than or equal to 40° in the afternoon. Nymphs showed a strong preference for the 30° shelter during both periods. The preferences of the animals in the morning and the afternoon were also compared. Eight percent to 15% of individuals did not show any shelter preference, which may be attributed to the limited selection time (see Table S1A (male), 2a (female), and 3a (nymphs) for statistical analysis results preferences in the morning and Table S1B (male), 2b (female), and 3b (nymph) for preferences in the afternoon). There was no significant difference in the shelter selection between the morning and afternoon for any of the experimental groups (supplementary 1c for males, 2c for females, and 3c for nymphs), suggesting that shelter preferences were not affected by the time of day.

### Shelter angle preference of individual adult German cockroaches after 24 h

**Figure 3:**
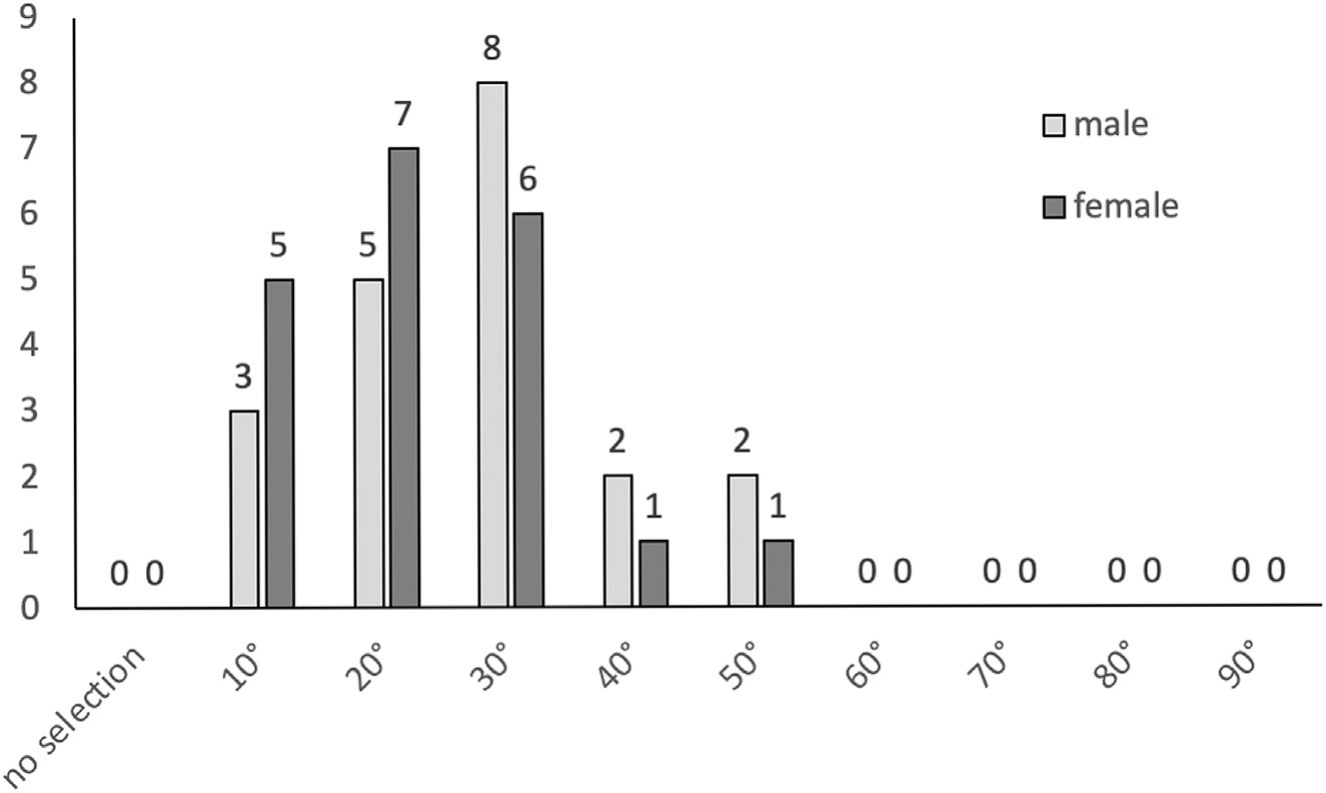
Shelter selection by individual German cockroaches after 24 hours One male or female German cockroach at a time was placed at the center of the acrylic cylinder for shelter selection. Copper shelters with angles of 10°, 20°, 30°, 40°, 50°, 60°, 70°, 80°, and 90° were evenly distributed inside the 60 cm cylinder at a distance of 18 cm from the center of the cylinder. The angle of the chosen copper shelter was recorded 24 h later, after which the cockroach was removed. X axis: the angle of the copper shelter; Y axis: numbers of cockroaches choosing a shelter.

After 24 h of shelter selection by individual German cockroaches, a large proportion chose to hide in shelters with an angle less than or equal to 30°. No cockroaches were found in shelters with an angle greater than or equal to 60° (Figure 3). Males showed a strong preference for shelters with a 30° angle (eight individuals out of 20), while females showed no significant preferences among shelters of 10°-30°. Five, seven, and six females were recorded in shelters with angles of 10°, 20°, and 30°, respectively (Figure 3). The Kendall rank correlation coefficient test confirmed that the preferences for both males and females were significant (p < 0.05), suggesting a strong preference for small shelter angles for in male and female adults (see Table S4A (male) and 4B (female)).

### Developmental trajectories of German cockroaches raised in shelters with angles of 30°, 90°, and 180°

Life tables for German cockroaches that developed in either a paper shelter or copper shelters with angles of 30°, 90°, and 180° were recorded. The lifespan of males was not significantly different in the paper shelter, 30 and 90°, while individuals that developed in 180° shelters showed extended lifespans (156.2 ± 35.4 days). Cockroaches that developed in shelters with large angles showed a more prolonged nymph stage (58.8 ± 13.3 and 63.1 ± 19.5 days for 90 and 180° shelters, respectively) than those that developed in the paper shelter (40.6 ± 15.4 days). In contrast, the duration of the adult stage showed no correlation with the angle of the shelter (Table 1). For female German cockroaches, individuals that developed in the 30° shelter showed a shorter lifespan (179.2 ± 38.2 days) than those that developed in the paper shelter (216.9 ± 37.5 days), while there was no significant difference between the paper shelter and 90° or 180° (Table 2). The duration of the nymph stage increased with the angle of the shelters, while there was no significant correlation between the lifespan of adults and the angle of the shelter. Cockroaches that developed in the paper shelter had the longest adult stage (175.3 ± 31.6 days), while those that developed in 30° shelters had the shortest adult stage (129.9 ± 23.2 days) (Table 2). Although cockroaches that developed in 180° shelters tended to have a long nymph stage (71.3 ± 14 days), the duration from hatching to fecundity was shorter than that of cockroaches raised in paper shelters (58.7 ± 2.5 days). The duration from full maturity to fecundity was also shorter (8.5 ± 0.7 days) in this shelter than in the paper shelter or the others (20.5-26.7 days) (Table 2). In addition, the fecundity rate of cockroaches that developed in 180° shelters was also significantly lower (19 ± 8.7%) than in those that developed in the paper shelter (80 ± 10 days) or the other shelters (77.7 ± 9.8 and 55.7 ± 9.2% for the 30 and 90° shelters, respectively). The percentage of nymphs that developed into the adult stage was counted as the survival rate. German cockroaches that developed in shelters with angles of 90 and 180° showed a significantly lower survival rate (61.7 ± 7.5%) than both the paper shelter (95 ± 5.5%) and those in 30° shelters (85 ± 10.5%) (Table 3).

**Table 1:** The lifespan, duration of the nymph stage, and duration of the adult stage in male German cockroaches that developed in shelters of different angles (Student’s *t* test between groups)

Treatment	Lifespan (days)	Duration of nymph stage (days)	Duration of adult stage (days)
paper shelter	127.6 ± 13.1a	40.6 ± 15.4a	87 ± 6.9c
30°	135.6 ± 32.2a	43.4 ± 15.4a	92.3 ± 24.2b
90°	135.6 ± 24.8a	58.8 ± 13.3b	76.8 ± 12.2a
180°	156.2 ± 35.4b	63.1 ± 19.5b	93.1 ± 20.9c

a, b, c: Multiple pairwise t tests between groups were performed. Entries in the same column with different letters are significantly different (p < 0.05).

**Table 2:** The lifespan, duration of the nymph stage, and duration of the adult stage in female German cockroaches that developed in shelters of different angles (Student’s *t* test between groups)

Treatments	Lifespan (days)	Duration of nymph stage (days)	Duration of adult sage (days)	Duration from hatching to fecundity (days)	Duration from full maturity to fecundity (days)	Fecundity (%)
paper shelter	216.9 ± 37.5b	40.5 ± 14a	175.3 ± 31.6a	58.7 ± 2.5b	26.7 ± 1.4days	80 ± 10c
30°	179.2 ± 38.2a	49.3 ± 17.4ab	129.9 ± 23.2a	58.7 ± 2.6b	23.7 ± 1.8c	77.7 ± 9.8c
90°	209.2 ± 41.5b	56.1 ± 21.5b	153.2 ± 25b	55.8 ± 3.8ab	20.5 ± 2.8b	55.7 ± 9.2b
180°	229.1 ± 43.7b	71.3 ± 14c	157.8 ± 32.9bc	54.5 ± 0.7a	8.5 ± 0.7a	19 ± 8.7a

a, b, c, d: Multiple pairwise t tests between groups were performed. Entries in the same column with different letters are significantly different (p < 0.05).

**Table 3:** Survival rates of German cockroaches that developed in shelters of different angles (Student’s *t* test between groups)

Treatments	Survival rate (%)
Control	95 ± 5.5a
30°	85 ± 10.5c
90°	61.7 ± 7.5b
180°	61.7 ± 7.5b

a, b, c: Multiple pairwise t tests between groups were performed. Entries in the same column with different letters are significantly different (p < 0.05).

Regarding the behavioral observations, cockroaches in the paper shelter and the 30° shelter were difficult to observe because most individuals were hidden by the shelter. Cockroaches in the 90° shelter stayed near the bend in the shelter roof, while those in 180° shelters aggregated along one corner of the copper square (Figure S3, second-to third-instar nymphs in the paper shelter; Figure S4, fourth-to fifth-instar nymphs in the paper shelter).

### High numbers of differentially expressed genes, DE-Gs, were identified at 2 days post-molting in both second- and sixth-instar nymphs

We collected samples at four-time points, i.e., 12 h post-molting and 2 days post-molting, in both second^−^ and sixth-instar nymphs to determine whether the shelter angle affected gene expression. Read yields per sample and mapping rates are shown in Table S5A. Read counts per gene and fragments per kilobase per million (FPKM) levels are shown in Tables S5B and S6. DE-Gs were identified by comparing the gene expression levels of cockroaches that developed in 30°, 90°, and 180° shelters with those of cockroaches that developed in the paper shelter. At 12 h post-molting, very few DE-Gs were identified in either the second- or sixth-instar nymphs for any of the three shelter angles (Table 3). However, effects of different shelter angles on gene expression were observed 2 days post-molting. For cockroaches that developed in the 30° shelter, a total of 346 (1.34%) and 297 (1.15%) DE-Gs were identified in the second- and sixth-instar nymphs, respectively. High numbers of DE-Gs were identified in cockroaches that developed in shelters with large angles (90 and 180°), but there was no linear correlation between the number of DE-Gs and the measure of the angle. The numbers of DE-Gs identified in cockroaches from 90° shelters were 1,277 (4.96%, second instar) and 445 (1.73%, sixth instar), and the corresponding numbers for 180° shelters were 989 (3.84%, second instar) and 445 (1.73%, sixth instar) (Table 4). The functions and gene IDs of the DE-Gs are shown in Table S7A-l.

**Table 4:** Numbers of differentially expressed genes in German cockroaches that developed in shelters of different angles in the comparison of 30°, 90°, and 180° to paper shelter

Number of DE-Gs						Percentage of DE-Gs (%)			
Angle	Expression trend	12 h post-molting		2 days post-molting		12 h post-molting		2 days post-molting	
		2nd instar	6th instar	2nd instar	6th instar	2nd instar	6th instar	2nd instar	6th instar
30°	Up	5	8	225	214	0.02	0.03	0.87	0.83
	Down	27	2	121	83	0.10	0.01	0.47	0.32
90°	Up	0	28	572	339	0.00	0.11	2.22	1.32
	Down	1	63	705	106	0.00	0.24	2.74	0.41
180°	Up	21	0	650	227	0.08	0.00	2.53	0.88
	Down	7	4	339	218	0.03	0.02	1.32	0.85
30°	total DE-Gs	32	10	346	297	0.12	0.04	1.34	1.15
90°	total DE-Gs	1	91	1277	445	0.00	0.35	4.96	1.73
180°	total DE-Gs	28	4	989	445	0.11	0.02	3.84	1.73

To more precisely evaluate the effect of the shelter angle, we further compared the gene expression profiles of 30°-90 and 180°. For second-instar nymphs, more DE-Gs were identified from 2-day-post-molting nymphs (680 and 490 DE-Gs for 90 and 180°) than 12-hour-post-molting nymphs (7 and 117 DE-Gs for 90 and 180°). For sixth-instar nymphs, the numbers of DE-Gs identified from 12 h post-molting and 2 days post-molting at 90 and 180° were 199 (12 h, 90°), 34 (12 h, 180°), 138 (2 days, 90°), and 395 (2 days, 180°) (Table 5).

**Table 5:** Numbers of differentially expressed genes in German cockroaches that developed in shelters of different angles in the comparison of 90 and 180°-30°

Angle	Expression trend	Number of DE-Gs				Percentage of DE-Gs (%)			
		12 h post-molting		2 days post-molting		12 h post-molting		2 days post-molting	
		2nd instar	6th instar	2nd instar	6th instar	2nd instar	6th instar	2nd instar	6th instar
90°	Up	7	70	239	53	0.03	0.27	0.93	0.21
	Down	0	129	441	85	0.00	0.50	1.71	0.33
180°	Up	95	5	332	219	0.37	0.02	1.29	0.85
	Down	22	29	158	176	0.09	0.11	0.61	0.68
90°	total DE-Gs	7	199	680	138	0.03	0.77	2.64	0.54
180°	total DE-Gs	117	34	490	395	0.45	0.13	1.90	1.54

### Gene ontology analysis of second-instar nymphs at 2 days post-molting

Gene ontology analysis was then performed to understand the functions of the DE-Gs. The GO results, including cellular component and molecular function terms, of all stages of cockroaches are shown in Table S8A-l (second-instar nymphs) and Table S9A-S9N (sixth-instar nymphs). For upregulated genes, the GO terms *cellular amide metabolic process* (GO:0043603) and *metabolic process* (GO:0008152) were identified in cockroaches that developed in the 30°, 90°, and 180° shelters. The GO terms *ATP metabolic process* (GO:0046034), *cellular respiration* (GO:0045333), *cellular nitrogen compound biosynthetic process* (GO:0044271), *mitochondrial ATP synthesis coupled electron transport* (GO:0042775), *mitochondrial electron transport*, and *NADH to ubiquinone* (GO:0006120) were found in cockroaches that developed in the 90 and 180° shelters. The terms *aerobic electron transport chain* (GO:0019646), *protein-containing complex assembly* (GO:0065003), *phosphorylation* (GO:0016310), and *ribosomal small subunit biogenesis* (GO:0042274) were found only in the group that developed in 90° shelters. In comparison, the terms *heterocycle metabolic process* (GO:0046483) and *ribonucleoprotein complex subunit organization* (GO:0071826) were found only in the group that developed in 180° shelters (Table S10A).

Regarding the GO terms with downregulated genes, *chitin-based cuticle development* (GO:0040003) was identified for the 90 and 180° shelters, while *homeostatic process* (GO:0042592), *larval development* (GO:0002164), *molting cycle, chitin-based cuticle* (GO:0007591), *regulation of tube architecture, open tracheal system* (GO:0035152), and *respiratory system development* (GO:0060541) were found only for the 90° shelters (Table S10B).

### Gene ontology analysis of sixth-instar nymphs at 2 days post-molting

Compared to the second-instar nymphs, relatively few DE-Gs and GO terms were found in the sixth-instar nymphs. For upregulated genes, very few terms were found to be shared among the different treatments. The terms *amide biosynthetic process* (GO:0043604), *cellular amide metabolic process* (GO:0043603), *cytoplasmic translation* (GO:0002181), *gene expression* (GO:0010467), *peptide biosynthetic process* (GO:0043043), and *translation* (GO:0006412) were identified in cockroaches from the 30 and 90° shelters. For cockroaches that developed in shelters with an angle of 180°, DE-Gs were enriched in the terms *aminoglycan metabolic process* (GO:0006022), *chitin-based cuticle development* (GO:0040003), and *chitin catabolic process* (GO:0006032). In comparison, the terms associated with the 30° shelters were *ATP metabolic process* (GO:0046034), *cellular nitrogen compound metabolic process* (GO:0034641), *energy derivation by oxidation of organic compounds* (GO:0015980), *generation of precursor metabolites and energy* (GO:0006091), and *organic substance metabolic process* (GO:0071704) (Table S10C).

Although 83 and 106 DE-Gs were found for the 30 and 90° shelters, respectively, functional terms were not identified. For cockroaches that developed in the 180° shelter, DE-Gs were enriched in the terms *chitin-based cuticle development* (GO:0040003), *imaginal disc morphogenesis* (GO:0007560), *instar larval or pupal morphogenesis* (GO:0048707), *molting cycle, chitin-based cuticle* (GO:0007591), *post-embryonic development* (GO:0009791), and *regulation of tube architecture, open tracheal system* (GO:0035152) (Table S10D).

### GO analysis of 90 and 180° compared to 30° at both the second and sixth instars

For the 90 and 180° shelters, fewer DE-Gs were identified than for the 30° shelter, and the number of annotated functional terms was also lower. GO terms could be identified only for the downregulated genes associated with 90 vs. 30° in 2-day-post-molting second-instar nymphs, 180 vs. 30° in 2-day-post-molting second-instar nymphs, and 90 vs. 30° in 12-hour-post-molting sixth-instar nymphs (see Table S11A-F for GO terms with FDR rate <0.05, Table S12A-S12O for GO terms of second-instar nymphs with p value < 0.05, Table S13A-S13I for GO terms of sixth-instar nymphs with p value <0.05). The GO terms *cuticle development* (GO:0042335), *chitin metabolic process* (GO:0006030), and *carbohydrate metabolic process* (GO:0005975) were identified for the downregulated DE-Gs of second-instar nymphs in the 90° shelter, while the terms *carbohydrate metabolic process* (GO:0005975), *energy reserve metabolic process* (GO:0006112), and *polysaccharide metabolic process* (GO:0005976) were identified for the downregulated DE-Gs of second-instar nymphs in the 180° shelter (Table S14). Fewer enriched terms were identified in sixth-instar nymphs; the enriched terms were *aminoglycan metabolic process* (GO:0006022), *chitin metabolic process* (GO:0006030), and *glucosamine-containing compound metabolic process* (GO:1901071).

## Discussion

Thigmotaxic behavior, also known as "wall-hugging," is common among vertebrates and invertebrates, especially in response to a novel environment (Treit and Fundytus, 1988; Simon et al., 1994; Durier and Rivault, 2003; Jeanson et al., 2003; Besson and Martin, 2004; Sharma et al., 2009; Schnörr et al., 2012). It enables animals to find shelter or escape routes in exploratory situations^6^. For cockroaches, thigmotaxis stimuli are received from the environment and the aggregation behavior of other individuals. Tactile stimuli generated from group effects have been extensively studied in cockroaches (Gadot et al., 1989; Lihoreau and Rivault, 2008), although the environmental factors affecting their development have received less attention. We used the shelter angle as a quantitative method to evaluate whether the degree of thigmotaxis generated by particular shelter characteristics affects German cockroaches' development, mortality, fecundity, and gene expression.

In the individual cockroach shelter selection trials, all individuals experienced a new environment and should, therefore, seek an edge feature or any other potential shelter. In a familiar environment, the exploratory behavior of German cockroaches can extend over all of the available surfaces. In contrast, a high level of tactile stimulus/edge-following behavior is observed when exploring a new environment (Durier and Rivault, 2003; Jeanson et al., 2003). Quick responses were observed in the 15-min shelter selection trial; only 7.5-15% of tested individuals remained open at the end of the trial. A strong preference for the 40° angle shelter was observed at all life stages and in both sexes. A similar tendency was also observed in the 24-h trials, except that all the tested individuals had hidden by the end of the trial. Comparing the shelter selection results of groups of cockroaches and individual adults in the 24-h trials, grouped adults showed a strong preference for shelters with an angle smaller than 30° (more than 95% of adults), while individual cockroaches, especially males (20%), could be found in the 40 and 50° shelters (Figure 3). Aggregation pheromones may affect shelter selection (Ledoux, 1945; Sakuma and Fukami, 1993; Planas-Sitjà et al., 2015), but the behavior of groups is also correlated with individual angular preferences (Planas-Sitjà and Deneubourg, 2018). Thus, the shelter selection experiments indicated an innate preference for sheltering in environments with angles less than or equal to 40°. Several studies suggest that German cockroaches prefer a certain range of harborage widths, and the preferences vary among developmental stage, gender, and gravid status. Nevertheless, the width is, in general, no larger than 13 mm (Berthold and Wilson, 1967; Koehler et al., 1994). This preference may share a similar rationale to our experiment but represent it in different manners.

The life tables further confirmed the benefits of this preference. Significant delays in nymph development and low fecundity rates were observed in cockroaches that developed in the 90 and 180° shelters. We cannot exclude the possibility that the texture of the copper plate may generate some effects that alter the development of cockroaches, as the surface texture affects the behavior of German cockroaches while depriving them of food and water (Berthold, 1967). Nevertheless, a high survival rate was observed in cockroaches raised in a 30° shelter, suggesting that the angle caused the difference. The physiological basis behind this delayed development is unclear, but a similar result has been observed in gregarious cockroaches reared in isolation. Tactile stimuli are crucial for the development of gregarious cockroaches, such as *B. germanica* and *Symploce pallens*. Individuals raised in isolation show delayed development, while those reared in groups show increased rates of nymphal development and oothecae production (Gadot et al., 1989; Lihoreau and Rivault, 2008). Insufficient environmental tactile stimuli seem to induce a stress response in German cockroaches and prolong the nymph stage (Gemeno et al., 2011). From the physiological perspective, prolongation of the nymph stage can be found in other species of paurometabolous insects when exposed to stressful conditions. In the brown planthopper, *Nilaparvata lugens*, exposure to their upper lethal temperature (ULT50) during the first nymphal instar increases the duration of the nymph stage and reduces the ultimate fecundity rate of adults (Piyaphongkul et al., 2012). In the western tarnished plant bug *Lygus hesperus*, nymphs that develop under a highly variable temperature regime take longer to develop than those reared at a constant temperature. Highly variable temperatures serve as stressors (Spurgeon and Brent, 2019). In the aphid *Sitobion avenae*, the nymph stages tend to be prolonged during periods of water deficit (Liu et al., 2018). There is also molecular evidence regarding delayed development in nymphs. In 2-day-post-molting second-instar nymphs in 90° shelters, GO terms related to larval development, molting cycle, and chitin-based cuticle were downregulated.

In contrast, in 2-day-post-molting sixth-instar nymphs in 180° shelters, imaginal disc morphogenesis, instar larval or pupal morphogenesis, and postembryonic development were downregulated. The GO terms of DE-Gs also reflected different developmental phases: the term "imaginal disc morphogenesis" relates to the change from larval to adult form (http://www.informatics.jax.org/vocab/gene_ontology/GO:0007560). In summary, shelters providing insufficient tactile stimuli appear likely to cause a stress response in German cockroach nymphs.

Insufficient tactile stimulus derived from the shelter environment also changed the energy requirement of cockroaches. The transcriptome analysis results showed that terms related to ATP metabolic process, mitochondrial ATP synthesis coupled electron transport, and mitochondrial electron transport were identified in second-instar nymphs that developed in 90 and 180° shelters and in sixth-instar nymphs that developed in 30° shelters. Energy consumption is related to insect locomotion type, active/resting state, and aggregation behavior (Tanaka et al., 1988; Reinhold, 1999). Upon exposure to stressors, energy consumption rises, and/or energy is reallocated to priority activities (Williams et al., 2012; Sinclair et al., 2013; Klepsatel et al., 2016). Energy reallocation can also impair reproductive system function and lower fecundity (Marshall and Sinclair, 2010). We observed changes in energy-related terms from a molecular perspective and a low fecundity rate from a physiological perspective. Stress induced by insufficient tactile stimulus may result in changes in energy consumption and further stress. It is also possible that additional energy requirements may lengthen the developmental time taken to reach the next instar and consequently prolong the duration of the nymphal period.

The angle of the shelter affects not only the development and fecundity of German cockroaches but also their survival rate. Compared to the paper shelter, a higher survival rate was observed for individuals that developed in shelters with angles of 90 and 180° (95 ± 5.5% for the paper shelter and 61.7 ± 7.5% for both 90 and 180° shelters) (Table 2). The life table for the German cockroaches was established based on a group of 20 nymphs so that the tactile stimulus generated from aggregation could be taken into account. The higher mortality rate in large-angle shelters suggests either that larger shelter angles produce some lethal effects or that nymphs cannot receive sufficient tactile stimulus from large-angle environments, resulting in a higher mortality rate. However, we cannot exclude the possibility that the group effect negatively impacts cockroaches hiding in large-angle shelters, although this seems very unlikely. From a molecular perspective, genes related to chitin-based cuticle development and the molting cycle were affected in second- and sixth-instar nymphs in shelters with angles of 90 and 180°, respectively. The change in chitin-related terms seems to be an indicator of insect exposure to stressors. The downregulation of the chitin metabolic process and chitin-based cuticle development may suggest that the structure, integrity, and even the cuticle thickness were affected. Compared to the paper shelter, we did not observe a higher rate of failure in molting. As cockroaches developed in the shelter with an angle of 30° show developmental status similar to that of the paper control, we then further used 30° as a comparison basis to more specifically emphasize the effect. The numbers of DE-Gs identified from each comparison were fewer than those from the paper control. It is unclear whether this difference was generated by the materials or by the shape of the shelter. A shelter of 30° was sufficient and seemed to be highly preferred by cockroaches in the shelter selection experiment. Fewer DE-Gs were observed in the comparison of 90 and 180°-30°, yet we could still observe functional terms related to cuticle development, carbohydrate metabolic processes, and energy reserve metabolic processes. These GO terms represented effects generated only from angles of shelter, as the effects from the texture of shelter or any other environmental stimuli can be eliminated during comparison. Thus, the effect of angle was confirmed to be correlated with cuticle development and metabolism. It was not possible to observe or evaluate the morphological changes in this study, but cockroach nymphs that developed in shelters with large angles might have developed weaker cuticle structures and may have been more vulnerable than those raised in paper shelters, resulting in a higher mortality rate. Further study of potential cuticle structural changes is required to explore this hypothesis.

In conclusion, German cockroaches showed a strong preference to hide in shelters with roof angles less than or equal to 40°. Groups of German cockroaches that developed in shelters with roof angles greater than or equal to 90° showed significant stress responses similar to those of isolated individuals without sufficient tactile stimuli resulting from aggregation. Life table and transcriptomic results revealed that German cockroaches forced to hide in unsuitable shelters suffered a prolonged duration of the nymph stage, high energy costs, low fecundity rate, and low survival rate, suggesting that environments without suitable shelters have a negative impact on the homeostasis of German cockroaches.

## STAR★Methods

### Key resources table

**Table:** 

REAGENT or RESOURCE	SOURCE	IDENTIFIER
**Biological samples**		

German cockroaches, *Blattella germanica*	ENTOMEK ENTERPRISE CORP	N/A

**Chemicals, peptides, and recombinant proteins**		

RNAeasy® Mini Kit	Qiagen	Cat. No. 74106
oligod(T)25 magnetic beads	New England BioLabs	S1419S
ProtoScript® II First Strand cDNA Synthesis Kit	New England BioLabs	E6560S
DNA polymerase I (*E. coli*)	New England BioLabs	M0209S
Rnase H	New England BioLabs	M0297S
NEBNext® Ultra™ II End Repair/dA-Tailing Module	New England BioLabs	E7546S
T4 DNA ligase	New England BioLabs	M0202S
Uracil-DNA Glycosylase (UDG)	New England BioLabs	M0280S
Q5® Hot Start High-Fidelity DNA Polymerase	New England BioLabs	M0493S
NEBNext® Ultra™ II Q5® Master Mix	New England BioLabs	M0544S
RNAClean XP	Beckman Coulter	A63987
Lithium dodecyl sulfate	Sigma-Aldrich	L9781-5G
Lithium Chloride	Sigma-Aldrich	203637-10G
Diethyl pyrocarbonate/DEPC (liquid)	Sigma-Aldrich	D5758-5ML
2-mercaptoethanol (liquid)	Sigma-Aldrich	M6250-10ML
100% EtOH	Sigma-Aldrich	32221-2.5L
UltraPure™ 1 M Tris-HCI Buffer, pH 7.5	Thermofisher	15567-027

**Deposited data**		

RNA-seq raw data	NCBI SRA archive	Accession ID: PRJNA771478

**Oligonucleotides**		

See Table S15 for RNA-seq PCR oligos list	Institute of Biotechnology, National Taiwan University	N/A

**Software and algorithms**		

bowtie 2	Langmead and Salzberg (2012)	http://bowtie-bio.sourceforge.net/bowtie2/index.shtml
eXpress	Roberts and Pachter (2013)	https://pachterlab.github.io/eXpress/overview.html
DESeq2	Love et al. (2014)	https://bioconductor.org/packages/release/bioc/html/DESeq2.html

**Other**		

German cockroach genome		GCA_000762945.2

### Resource availability

#### Lead contact

Further information and requests for resources and reagents should be directed to and will be fulfilled by the lead contact, En-Cheng Yang, ecyang@ntu.edu.tw.

#### Materials availability

This study did not generate new unique reagents.

### Experimental models and subject details

German cockroaches were from ENTOMEK ENTERPRISE CORP. Different developmental stages and genders of German cockroaches were used in this report.

### Method details

#### German cockroach

German cockroaches were raised in a plastic container (23 × 15 × 10 cm). A 10 cm × 10 cm paper sheet was folded into a zig-zag step shape with ten flat surfaces and laid horizontally to provide shelter for the cockroaches. Pinholes were punched into the lid of the container for ventilation. The inner side of the container was coated with polytetrafluoroethylene (PTFE) to prevent the cockroaches from escaping. The box was kept in a room at 27 ± 1°C, with 30–50% humidity and a daily light-dark cycle of 12 h:12 h. Dry cat food and fresh water were replenished daily.

#### Shelter angle preference of German cockroaches

##### Environment design

The test environment comprised a lidded acrylic cylinder (inside diameter 59 cm × height 11.5 cm; outside diameter 60 cm × height 20 cm) (Figure S1A). The cylinder was kept at 27 ± 1°C, with a humidity of 30–50% and a light/dark cycle of 12 h:12 h. The inner side of the cylinder was coated with PTFE to prevent the experimental animals from climbing the sides or corners of the cylinder, and the bottom of the cylinder was covered with white paper. Copper plates measuring 10 × 10 × 0.03 cm were bent to create tent-shaped shelters with angles of 10°, 20°, 30°, 40°, 50°, 60°, 70°, 80°, and 90°. The nine copper shelters with different fold angles were distributed evenly inside the acrylic cylinder at a distance of 18 cm from the center of the cylinder (Figure S1B), with the opening of the shelter directed toward the center of the cylinder. Each trial was performed for 24 hours, with a daily light-dark cycle of 12 h:12 h, after which the test cockroaches were removed. This cylinder will be termed the ‘60 cm-cylinder’ hereafter to distinguish it from other experimental conditions.

#### Shelter angle preference of groups of nymph, adult male, and adult female German cockroaches after 24 hours

The shelter angle preference was tested in three different groups of cockroaches: nymphs, adult males, and adult females. Twenty 1-day-old nymphs were used for the nymph assay, and 10 males or 10 females for the adult assay were placed at the center of the 60-cm cylinder and left to select their preferred shelters. Each trial was performed for 24 hours, with a daily light-dark cycle of 12 h:12 h, after which the test cockroaches were removed. To ensure that no pheromone residues remained on the cylinder surface, all copper shelters and the cylinder were rinsed with acetone between trials (Macron 2435-10). The paper floor was replaced with clean paper. The locations of the variously angled copper shelters were randomly rearranged before every experiment. Each trial was repeated six times to test a total of 120 nymphs, 60 adult males and 60 adult females. The selection was analyzed by Student’s t test (1 tail, two-sample equal variance) to confirm whether the preferences were significant.

#### Shelter angle preference of individual nymph, adult male, and adult female German cockroaches after 15 minutes

One healthy 1-day-old nymph or one 27- to 37-day-old male or female German cockroach was placed at the center of the 60-cm cylinder for shelter selection. The cylinder surface and all copper shelters were rinsed with acetone to remove pheromone residues (Macron 2435-10), and the paper floor was replaced with clean paper. The experiments were performed in the morning (between 6:00 and 12:00) and the afternoon (between 12:00 and 18:00). Twenty nymphs, 10 males, and 10 females were tested in the morning and afternoon, making a total of 40 nymphs, 20 males and 20 females tested. Each trial was performed for 15 minutes, after which the test cockroach was removed.

#### Shelter angle preference of individual adult German cockroaches after 24 hours

One healthy 27–37-day-old adult male or female German cockroach was placed at the center of the 60-cm cylinder for shelter selection. After 24 h, the angle of the chosen copper shelter was recorded, and the cockroach was removed. The cylinder walls and all copper shelters were rinsed with acetone (Macron^TM^ 2435-10) to remove pheromone residues, and the paper floor was replaced. A total of 20 males and 20 females were tested.

#### Life table of German cockroaches that developed in shelters with angles of 30°, 60°, and 180°

Life tables were established based on cockroaches housed in a clean glass cylinder (inside diameter 11 cm × height 11.5 cm; outside diameter 12 cm × height 12 cm; wall thickness: 0.5 cm) (see Figure S2 for environment design). A piece of gauze was fixed over the open end of the cylinder with a rubber band to ensure air circulation and prevent the cockroaches from escaping. The inside of the cylinder was coated with PTFE to prevent the experimental animals from climbing the side and corners of the cylinder. A 10 × 10 × 0.03 cm copper square was folded to create shelters with angles of 30° or 90°. In addition, an 8 × 8 × 0.03 cm copper square was left unbent to create a 180° shelter. The gaps between the copper squares and the bottom of the cylinder were sealed with Vaseline to prevent nymphs from squeezing through the gap. A piece of flattened cotton was soaked with water to provide a water supply. Pulverized dry cat food was supplied every other day. Paper shelter cockroaches were raised in a cylinder with a 10 × 10 cm sheet of white paper folded into a step-shaped zig-zag with 10 flat surfaces (resulting in an angle of 90° ± 15° between surfaces); this folded paper was laid horizontally as a shelter. Twenty newly hatched larvae were placed into the cylinders with a copper shelter with an angle of either 30°, 90°, or 180° so that the nymphs would develop in a consistent environment. There were three replicates for each shelter angle; hence, a total of 60 individuals were tested with each angle of shelter. Fresh food and water were provided daily. Life tables were recorded daily. The sex of the cockroaches was recorded once they had reached adulthood.

The 2^nd^- and 6^th^-instar cockroach nymphs were collected 12 hours and 2 days post-molting, respectively. Three biological replicates were collected, each of five individuals, making 15 individuals per age per shelter angle in total. Collected samples were stored in RNAlater® at −20°C for subsequent RNA extraction and RNA-seq library preparation.

#### RNA extraction and RNA-seq library preparation

The sampled cockroaches were finely ground in RLT buffer (supplemented with 1% 2-mercaptoethanol), vortexed for 30 seconds, and then chilled on ice for 10 minutes. The solution was then spun at 14,000 rpm to pellet the tissues. The supernatant was moved to a new 1.5 ml tube, and RNA was extracted using an RNAeasy® Mini Kit (Qiagen). The RNA quality was examined on an electrophoresis gel and then quantified using a Nanodrop 2000 (Thermo Scientific). At least 20 μg of total RNA/sample was used to prepare a strand-specific RNA-seq library.

Strand-specific RNA-seq libraries were constructed following Zhong et al. (2011). Briefly, polyA RNA was enriched twice with Oligo d(T)25 Magnetic Beads (NEB) and then heated to simultaneously elute and fragment polyA RNA in ProtoScript® II Reaction Mix (2×) in the presence of a random hexamer and oligo dTVN (NEB). First-strand cDNA was synthesized using a ProtoScript II First Strand cDNA synthesis kit (NEB) at 45°C for 45 minutes. Second strand cDNA was prepared using DNA polymerase I (NEB) and RNase H (NEB) supplemented with dUTP mix (2 mM dATP, 2 mM dCTP, 2 mM dGTP mix, and 4 mM dUTP, ThermoFisher). After end-repair and dA-tailing, double-stranded cDNA was ligated with a Y-shape Illumina adapter. Products were treated with uracil DNA glycosylase and then amplified with the index primer sets (see Table S15 for PCR oligo sequences). Sequencing was performed on the Illumina NovaSeq 6000 platform.

#### Gene expression estimation and DEG (differentially expressed gene) analysis

The German cockroach genome GCA_000762945.2 was used in this study. After removal of duplicate, incomplete, and non-ATG-initiated CDSs, a total of 25,732 reference CDSs were used for gene expression analysis. With the default setting, raw reads were mapped against the CDS using bowtie 2 (Langmead and Salzberg, 2012). Gene expression levels were estimated using eXpress (Roberts and Pachter, 2013). To evaluate whether the different shelter angles affected gene expression, DEGs were identified by comparing the gene expression profiles of cockroaches that developed in shelters with angles of 30°, 90°, and 180° with those of the paper shelter. To be more specific, the gene expression profiles of 90 and 180° were also compared with that of 30° to confirm the effect of the angle. DEGs were identified using Bioconductor DESeq2 (v.1.12.4; Bioconductor 3.7), followed by a Wald test to estimate the false discovery rate (FDR) (Love et al., 2014). Genes with FDR rate < 0.05 and fold change ≥ 2 folds (log_2_ fold change value ≥ 1 or ≤ −1) were considered differentially expressed.

DEG annotation was then performed by BLASTing against the landmark database (https://blast.ncbi.nlm.nih.gov/smartblast/smartBlast.cgi?CMD=Web&PAGE_TYPE=BlastDocs). Gene Ontology (GO) term analysis was performed using the online analysis website Gene Ontology (http://geneontology.org/) for functional enrichment and classification.

### Quantification and statistical analysis

The statistical analysis for the individual cockroach shelter selection was examined with the Kendall rank correlation coefficient. To confirm whether the shelter preference was different in the morning and in the afternoon, the shelter selection results of individual cockroaches between the morning and afternoon were also compared using the Mann–Whitney U test. For grouped cockroach shelter selection, development duration and survival rate of nymphs developed in the shelter with 30°, 90°, 180°, Student’s t test was performed. For all analysis results, a p value <0.05 was considered significant.

## Data Availability

The datasets have been uploaded to SRA and are publicly available at 2022-10-31 or the date of publication. Accession to cite for these SRA data: PRJNA771478.

## Supplemental information

Document S1. Figures S1–S4

Table S1. Statistical analysis of male cockroach shelter preference in 15 min A. Statistical analysis of single male cockroach shelter preference in 15 min during AM, related to STAR Methods. B. Statistical analysis of single male cockroach shelter preference in 15 min during PM, related to STAR Methods. C. Statistical analysis of single male cockroach shelter preference in 15 min. The preferences in the AM and PM were compared, related to STAR Methods.

Table S2. Statistical analysis of female cockroach shelter preference in 15 min A. Statistical analysis of single female cockroaches’ shelter preference in 15 min during AM, related to STAR Methods. B. The statistical analysis of single female German cockroach shelter preference in 15 min during PM, related to STAR Methods. C. The statistical analysis of single female German cockroach shelter preference in 15 min. The preferences in the AM and PM were compared, related to STAR Methods.

Table S3. Statistical analysis of cockroach nymph shelter preference in 15 min A. Statistical analysis of single cockroach nymph shelter preference in 15 min during AM, related to STAR Methods. B. Statistical analysis of single cockroach nymph shelter preference in 15 min during PM, related to STAR Methods. C. Statistical analysis of single cockroach nymph shelter preference in 15 min. The preferences in the AM and PM were compared, related to STAR Methods.

Table S4. Statistical analysis of male and female cockroach shelter preference in 24 hours A. Statistical analysis of male cockroaches’ shelter preference in 24 h, related to STAR Methods. B. Statistical analysis of female cockroaches’ shelter preference in 24 h, related to STAR Methods.

Table S5. The RNA-seq reads yield and read count A. The RNA-seq reads yield, related to Table 4. B. Read counts of genes, related to Table 4.

Table S6. Fragments per kilobase per million (FPKM) level for genes of *B. germanica* developed in different angles of shelters, related to Table 4

Table S7. Functions of DEGs for 2^nd^ and 6^th^ instar nymph A. DE-Gs functions of 2nd instar *B. germanica*, in 30° at 12 h post-molting, related to Table 4. B. DE-Gs functions of 2nd instar *B. germanica*, in 90° at 12 h post-molting, related to Table 4. C. DE-Gs functions of 2nd instar *B. germanica*, in 180° at 12 h post-molting, related to Table 4. D. DE-Gs functions of 2nd instar *B. germanica*, in 30° at 2-day post-molting, related to Table 4. E. DE-Gs functions of 2nd instar *B. germanica*, in 90° at 2-day post-molting, related to Table 4. F. DE-Gs functions of 2nd instar *B. germanica*, in 180° at 2-day post-molting, related to Table 4. G. DE-Gs functions of 2nd instar *B. germanica*, in 30° at 12 h post-molting, related to Table 4. H. Functions of DE-Gs of sixth instar *B. germanica*, in 90° at 12 h post-molting, related to Table 4. I. Functions of DE-Gs of sixth instar *B. germanica*, in 180° at 12 h post-molting, related to Table 4. J. Functions of DE-Gs of sixth instar *B. germanica*, in 30° at 2-day post-molting, related to Table 4. K. Functions of DE-Gs of sixth instar *B. germanica*, in 90° at 2-day post-molting, related to Table 4. L. Functions of DE-Gs of sixth instar *B. germanica*, in 180° at 2-day post-molting, related to Table 4.

Table S8. Molecular Function and Cellular Component results of 2^nd^ instar cockroach nymphs A. Cellular component of up-regulated genes of 2-day post molting, 2nd instar *B. germanica* nymphs, in 30°, related to Table 4. B. GMolecular function of up-regulated genes of 2-day post molting, 2nd instar *B. germanica* nymphs, in 30°, related to Table 4. C. Cellular component of down-regulated genes of 2-day post molting, 2nd instar *B. germanica* nymphs, in 30°, related to Table 4. D. Molecular function of down-regulated genes of 2-day post molting, 2nd instar *B. germanica* nymphs, in 30°, related to Table 4. E. Cellular component of up-regulated genes of 2-day post molting, 2nd instar *B. germanica* nymphs, in 90°, related to Table 4. F. Molecular function of up-regulated genes of 2-day post molting, 2nd instar *B. germanica* nymphs, in 90°, related to Table 4. G. Cellular component of down-regulated genes of 2-day post molting, 2nd instar *B. germanica* nymphs, in 90°, related to Table 4. H. Molecular function of down-regulated genes of 2-day post molting, 2nd instar *B. germanica* nymphs, in 90°, related to Table 4. I. Cellular component of up-regulated genes of 2-day post molting, 2nd instar *B. germanica* nymphs, in 180°, related to Table 4. J. GMolecular function of up-regulated genes of 2-day post molting, 2nd instar *B. germanica* nymphs, in 180°, related to Table 4. K. Cellular component of down-regulated genes of 2-day post molting, 2nd instar *B. germanica* nymphs, in 180°, related to Table 4. L. Molecular function of down-regulated genes of 2-day post molting, 2nd instar *B. germanica* nymphs, in 180°, related to Table 4.

Table S9. Molecular Function and Cellular Component results of 6^th^ instar cockroach nymphs A. Cellular component of down-regulated genes of 12-h post molting, 6th instar *B. germanica* nymphs, in 90°, related to Table 4. B. Molecular function of down-regulated genes of 12-h post molting, 6th instar *B. germanica* nymphs, in 90°, related to Table 4. C. Gene Ontology analysis of the cellular component of up-regulated genes of 2-day post molting, 6th instar *B. germanica* nymphs, in 30°, related to Table 4. D. Gene Ontology analysis of the molecular function of up-regulated genes of 2-day post molting, 6th instar *B. germanica* nymphs, in 30°, related to Table 4. E. Gene Ontology analysis of the cellular component of down-regulated genes of 2-day post molting, 6th instar *B. germanica* nymphs, in 30°, related to Table 4. F. Gene Ontology analysis of the molecular function of down-regulated genes of 2-day post molting, 6th instar *B. germanica* nymphs, in 30°, related to Table 4. G. Gene Ontology analysis of the cellular component of up-regulated genes of 2-day post molting, 6th instar *B. germanica* nymphs, in 90°, related to Table 4. H. Molecular function of up-regulated genes of 2-day post molting, 6th instar *B. germanica* nymphs, in 90°, related to Table 4. I. Cellular component of down-regulated genes of 2-day post molting, 6th instar *B. germanica* nymphs, in 90°, related to Table 4. J. Molecular function of down-regulated genes of 2-day post molting, 6th instar *B. germanica* nymphs, in 90°, related to Table 4. K. Cellular component of up-regulated genes of 2-day post molting, 6th instar *B. germanica* nymphs, in 180°, related to Table 4. L. Molecular function of up-regulated genes of 2-day post molting, 6th instar *B. germanica* nymphs, in 180°, related to Table 4. M. Cellular component of down-regulated genes of 2-day post molting, 6th instar *B. germanica* nymphs, in 180°, related to Table 4. N. Molecular function of down-regulated genes of 2-day post molting, 6th instar *B. germanica* nymphs, in 180°, related to Table 4.

Table S10. Biological Process results of 2^nd^ and 6^th^ instar cockroach nymphs A Biological process of up-regulated genes of 2nd instar *B. germanica* , related to Table 4. B. Biological process of down-regulated genes of 2nd instar *B. germanica* , related to Table 4. C. Biological process of up-regulated genes of 6th instar *B. germanica*, related to Table 4 D. Biological process of down-regulated genes of 6th instar *B. germanica* , related to Table 4.

Table S11. GO results of 90° vs 30° and 180 vs 30° (FDR< 0.05) A. Biological process of down-regulated genes of 2-day post-molting, 2nd instar *B. germanica* nymphs, 90° vs 30°, related to Table 5. B. Cellular component of down-regulated genes of 2-day post-molting, 2nd instar *B. germanica* nymphs, 90° vs 30°, related to Table 5. C. Molecular function of down-regulated genes of 2-day post-molting, 2nd instar *B. germanica* nymphs, 90° vs 30°, related to Table 5. D. Biological process of down-regulated genes of 2-day post-molting, 2nd instar *B. germanica* nymphs, 180° vs 30°, related to Table 5. E. Molecular function of down-regulated genes of 2-day post-molting, 2nd instar *B. germanica* nymphs, 180°vs 30°, related to Table 5. F. Biological process of down-regulated genes of 12-h post-molting, 6th instar *B. germanica* nymphs, 90° vs 30°, related to Table 5.

Table S12. GO results of 90° vs 30° and 180 vs 30°of 2^nd^ instar nymph (p value < 0.05) A. Biological process of up-regulated genes of 12-hour-post-molting, 2nd instar *B. germanica* nymphs,180° vs 30°. Terms with p < 0.05 were shown, related to Table 5. B. Biological process of up-regulated genes of 2-day-post-molting, 2nd instar *B. germanica* nymphs, 90° vs 30°. Terms with p < 0.05 were shown, related to Table 5. C. Biological process of down-regulated genes of 2-day-post-molting, 2nd instar *B. germanica* nymphs., 90° vs 30°. Terms with p < 0.05 were shown, related to Table 5. D. Biological process of up-regulated genes of 2-day-post-molting, 2nd instar *B. germanica* nymphs, 180° vs 30°. Terms with p < 0.05 were shown, related to Table 5. E. Biological process of down-regulated genes of 2-day-post-molting, 2nd instar *B. germanica* nymphs, 180° vs 30°. Terms with p < 0.05 were shown, related to Table 5. F. Molecular function of up-regulated genes of 12-hour-post-molting, 2nd instar *B. germanica* nymphs180° vs 30°. Terms with p < 0.05 were shown, related to Table 5. G. Molecular function of up-regulated genes of 2-day-post-molting, 2nd instar *B. germanica* nymphs, 90° vs 30°. Terms with p < 0.05 were shown, related to Table 5. H. Molecular function of down-regulated genes of 2-day-post-molting, 2nd instar *B. germanica* nymphs, 90° vs 30°. Terms with p < 0.05 were shown, related to Table 5. I. Molecular function of up-regulated genes of 2-day-post-molting, 2nd instar *B. germanica* nymphs, 180° vs 30°. Terms with p < 0.05 were shown, related to Table 5. J. Molecular function of down-regulated genes of 2-day-post-molting, 2nd instar *B. germanica* nymphs, 180° vs 30°. Terms with p < 0.05 were shown, related to Table 5.

Table 13. GO results of 90° vs 30° and 180 vs 30°of 6^th^ instar nymph (p value < 0.05) A. Biological process of down-regulated genes of 12-hour-post-molting, 6th instar *B. germanica* nymphs, 90° vs 30°. Terms with p < 0.05 were shown, related to Table 5. B. Biological process of up-regulated genes of 2-day-post-molting, 6th instar *B. germanica* nymphs, 180°vs 30°. Terms with p < 0.05 were shown, related to Table 5. C. Biological process of down-regulated genes of 2-day post-molting, 6th instar *B. germanica* nymphs, 180° vs 30°. Terms with p < 0.05 were shown, related to Table 5. D. Molecular function of down-regulated genes of 12-h post-molting, 6th instar *B. germanica* nymphs, 90°vs 30°. Terms with p < 0.05 were shown, related to Table 5. E. Molecular function of up-regulated genes of 2-day post-molting, 6th instar *B. germanica* nymphs, 180° vs 30°. Terms with p < 0.05 were shown, related to Table 5. F. Molecular function of down-regulated genes of 2-day post-molting, 6th instar *B. germanica* nymphs, 180° vs 30°. Terms with p < 0.05 were shown, related to Table 5. G. Cellular component of down-regulated genes of 12-h post-molting, 6th instar *B. germanica* nymphs, 90° vs 30°. Terms with p < 0.05 were shown, related to Table 5. H. Cellular component of up-regulated genes of 2-day post-molting, 6th instar *B. germanica* nymphs, 180° vs 30°. Terms with p < 0.05 were shown, related to Table 5. I. Cellular component of down-regulated genes of 2-day post-molting, 6th instar *B. germanica* nymphs, 180° vs 30°. Terms with p < 0.05 were shown, related to Table 5.

Table S14. GO analysis results of DE-Gs of second and sixth instar German cockroaches developed in shelter with angles of 90 and 180 to that of 30. GO with false discovery rate <0.05 were listed, related to Table 5

Table S15. RNA-seq PCR oligos list, related to STAR Methods
