# Supplementary material for: The impact of thigmotaxis deprivation on the development of the German cockroach (Blattella germanica)
Source: iScience. 2022 Jul 20;25(8):104802. doi: 10.1016/j.isci.2022.104802 (PMC9385682; doi:10.1016/j.isci.2022.104802)
Supplement: Document S1. Figures S1–S4 [file mmc1.pdf]

iScience, Volume *25*

Supplemental information

The impact of thigmotaxis deprivation on the development of the German cockroach (*Blattella germanica*)

Yun-Ru Chen, De-Wei Li, Hsin-Ping Wang, Shih-Shun Lin, and En-Cheng Yang

Supplementary Figure 1. Environment design for shelter selection, related to STAR Methods

A

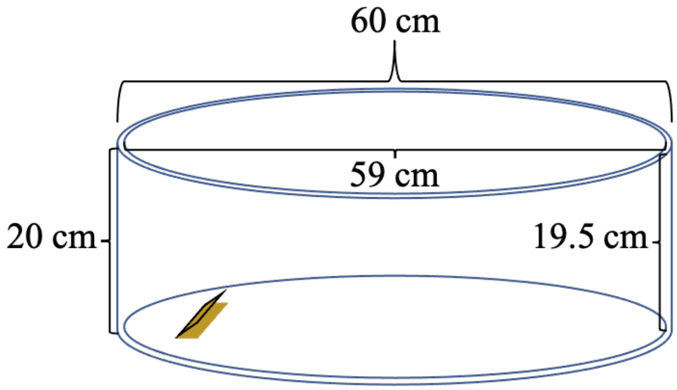

B

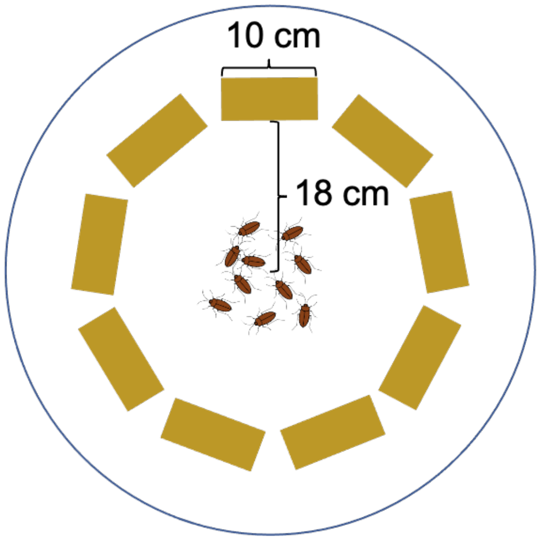

Supplementary Fig. 1. The environment design for shelter selection. **A.** The structure of the acrylic cylinder. Inner: rim × height = 59 cm ×11.5 cm; outside diameter × height = 60 cm × 20 cm. **B.** For shelter selection, a piece of 10 cm × 10 cm × 0.03 cm copper square was folded to create the environmental angels including 10°, 20°, 30°, 40°, 50°, 60°, 70°, 80°, and 90°. The nine copper shelters with different fold angles were distributed evenly inside the acrylic cylinder, at a distance of 18 cm from the center of the cylinder. The opening of the shelters was directed toward the center of the cylinder.

Supplementary Figure 2. Environment design for life table., related to STAR Methods

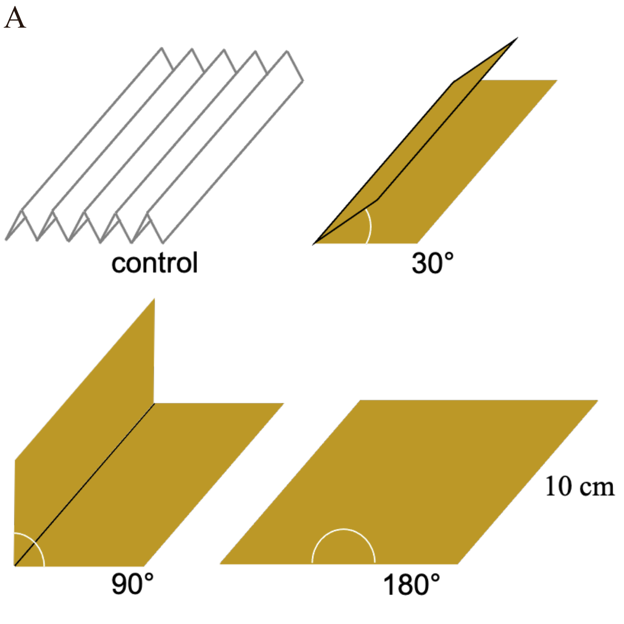

Supplementary Fig. 2. The environment design for life table. **A.** A piece of ladder-shape-folded paper was used as control. For angel treatment, a piece of 10 cm × 10 cm × 0.03 cm copper square was folded to create the environmental angels including 30°, 90°, and 180°. **b.** The angel experiment was set up in a in a clean glass cylinder, with inner: rim 11 cm, height 11.5; outside diameter: 12 cm, height 12 cm; wall: 0.5 cm. Inner side of the cylinder was coated with polytetrafluoroethylene (PTFE) to avoid the escaping of cockroaches. Slit between the copper square and the bottom of the cylinder was sealed with Vaseline to avoid nymphs in contact with the slit.

Supplementary Figure 3. Second to third instar nymphs distribution in an environment with specific angle of shelter, related to STAR Methods

A. Control

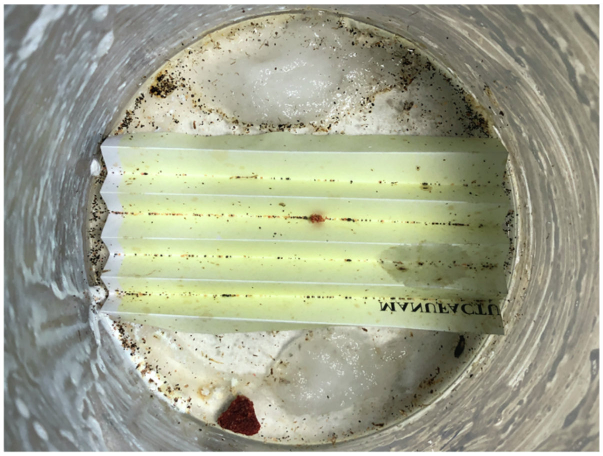

B. 30°

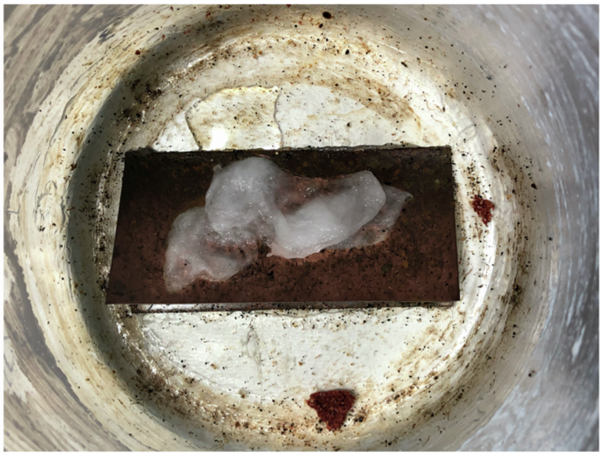

C. 90°

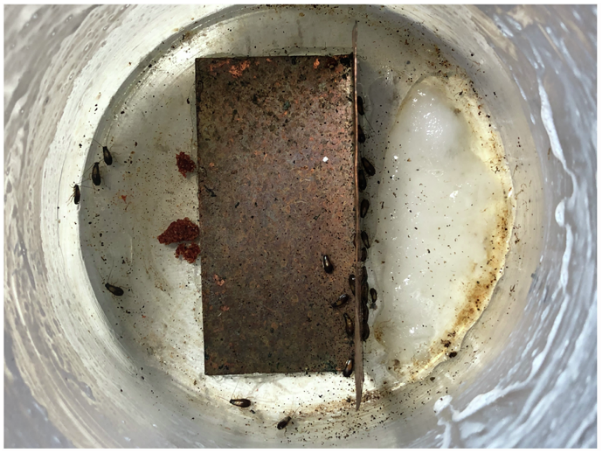

D. 180°

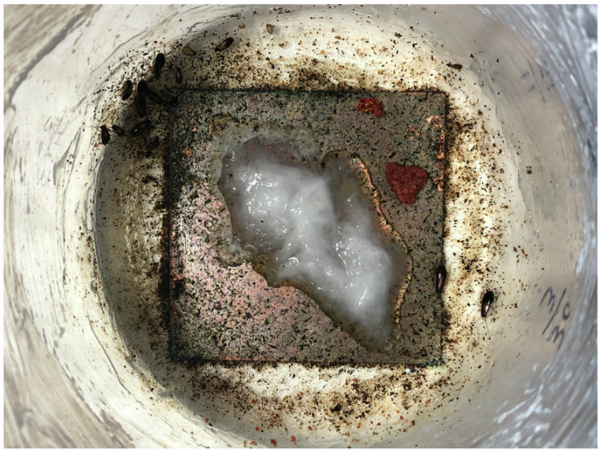

Supplementary Fig. 3. The distribution of 2nd to 3rd instar (in control) German cockroaches nymphs in cilynder with shelter angles of A. control, B. 30°, C. 90°, and D. 180°.

Supplementary Figure 4. Fourth to fifth instar nymphs distribution in an environment with specific angle of shelter, related to STAR Methods

A. Control

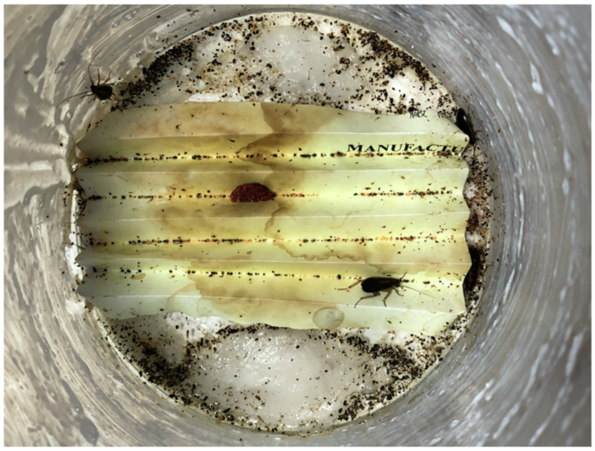

B. 30°

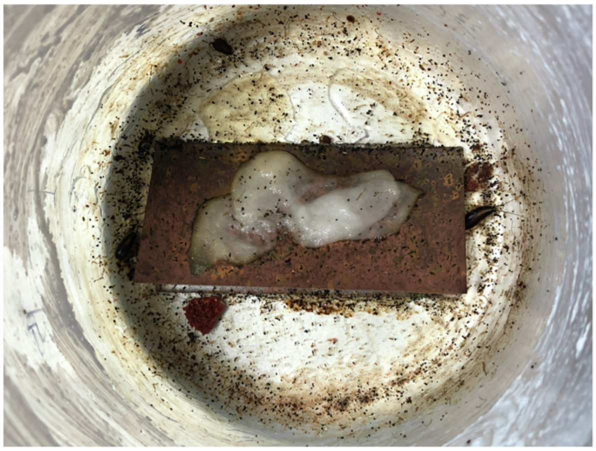

C. 90°

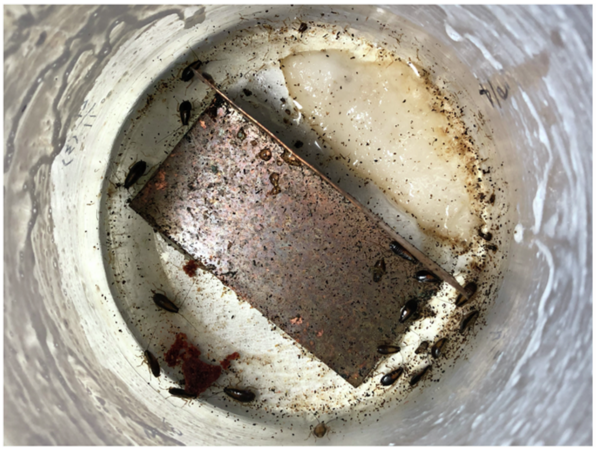

D. 180°

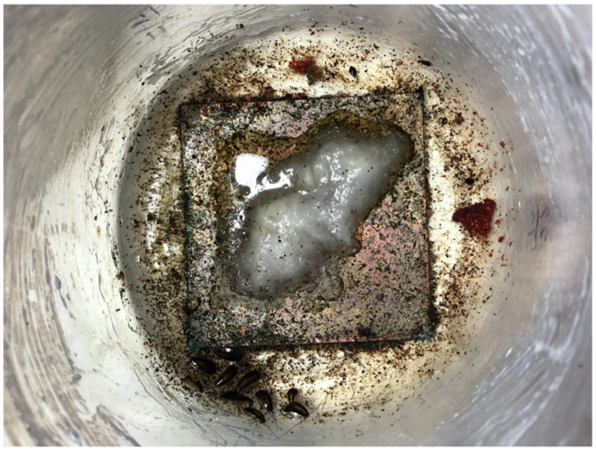

Supplementary Fig. 4. The distribution of 4th to 5th instar (in control) German cockroaches nymphs in cilynder with shelter angles of A. control, B. 30°, C. 90°, and D. 180°.
